# The genome sequence of the drosophilid fruit fly, *Drosophila phalerata *(Meigen, 1830)

**DOI:** 10.12688/wellcomeopenres.20634.1

**Published:** 2024-02-19

**Authors:** Darren J. Obbard, Darwin Tree of Life Barcoding collective, Wellcome Sanger Institute Tree of Life Management, Samples and Laboratory team, Wellcome Sanger Institute Scientific Operations: Sequencing Operations, Wellcome Sanger Institute Tree of Life Core Informatics team, Tree of Life Core Informatics collective, Darwin Tree of Life Consortium

**Affiliations:** 1 Institute of Ecology and Evolution, The University of Edinburgh, Edinburgh, Scotland, UK

**Keywords:** Drosophila phalerata, drosophilid fruit fly, genome sequence, chromosomal, Diptera

## Abstract

We present a genome assembly from an individual male *Drosophila phalerata* (drosophilid fruit fly, Arthropoda; Insecta; Diptera; Drosophilidae). The genome sequence is 223.9 megabases in span. Most of the assembly is scaffolded into 7 chromosomal pseudomolecules, including the X and Y sex chromosomes. The mitochondrial genome has also been assembled and is 16.14 kilobases in length. Gene annotation of this assembly on Ensembl identified 18,973 protein coding genes.

## Species taxonomy

Eukaryota; Metazoa; Eumetazoa; Bilateria; Protostomia; Ecdysozoa; Panarthropoda; Arthropoda; Mandibulata; Pancrustacea; Hexapoda; Insecta; Dicondylia; Pterygota; Neoptera; Endopterygota; Diptera; Brachycera; Muscomorpha; Eremoneura; Cyclorrhapha; Schizophora; Acalyptratae; Ephydroidea; Drosophilidae; Drosophilinae; Drosophilini; *Drosophila*; *Drosophila*; *quinaria* group; *Drosophila phalerata* (Meigen, 1830) (NCBI:txid7283).

## Background

**Figure 1.**
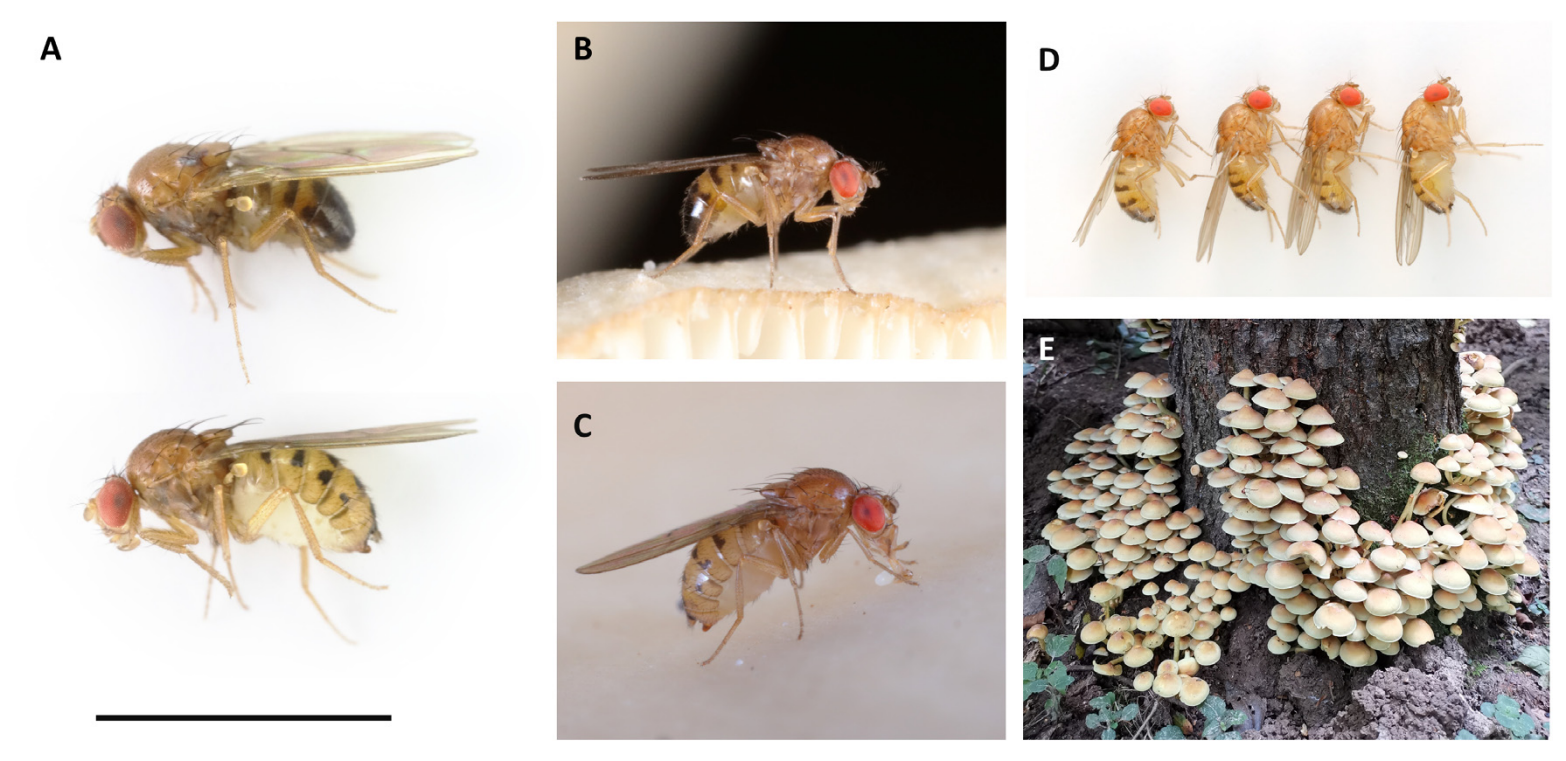
**A**: Male (above) and female (below) *Drosophila phalerata* presented with a 3 mm scale bar. **B** and **C**: Male and female *Drosophila phalerata*, respectively, photographed on oyster mushrooms ( *Pleurotus* spp.) in the Hermitage of Braid, Edinburgh, Scotland. **D**: The four lab-reared brothers selected for sequencing. Sample SAMEA12110456 (left) was used for HiC sequencing, and sample SAMEA12110457 (second left) was used for PacBio sequencing. **E**: The sulphur tuft fungus ( *Hypholoma fasciculare*) from which the mother of the sequenced flies was collected (Penns in the Rocks Estate, East Sussex, England; 51.093N,0.1698E).

*Drosophila phalerata* Meigen,1830 is a small to medium sized (2.2–3.0 mm) yellow-brown drosophilid ‘fruit fly’ ( Figure 1A–D), distantly related to the laboratory model *Drosophila melanogaster*. It is one of around 30 British and Irish species of *Drosophila* ( Chandler, 2023) and, like most its close relatives, it is a specialist fungus breeder ( Scott Chialvo *et al.*, 2019). *Drosophila phalerata* is among the most abundant fungus-breeding drosophilids in the UK ( Shorrocks, 1977), and is not thought to be threatened. The adults are easily collected or reared from many fungi, including *Phallus impudicus*, *Polyporus squamosus*, *Amanita rubescens*, *Pluteus cevinus* and *Tricholomopsis platyphylla* ( Charlesworth & Shorrocks, 1980; Shorrocks & Charlesworth, 1980). They are also attracted to decaying plant material ( Offenberger & Klarenberg, 1992) and can be collected in smaller numbers from yeasted fruit bait (e.g. Basden, 1955).

This species is broadly distributed in wooded areas across Europe, from the Azores and Madeira in the south and west, to Finland in the North, and Iran in the east ( Bächli, 2023). In the UK, the adults are most active from May to October ( GBIF Secretariat, 2023), but can be caught at any time of year ( Basden, 1955). Egg to adult development time is thought to be around 25 to 32 days in the field, and there are likely to be 4 to 5 generations in a season – although larval development is faster at higher temperatures ( Driessen *et al.*, 1989). Adults that eclose late in the year enter a daylength-induced diapause over winter, and emerge in the following spring ( Muona & Lumme, 1981). *Drosophila phalerata* has been studied for both its courtship behaviour (e.g. Hedlund *et al.*, 1996; Neems *et al.*, 1997), and its interaction with parasitoids and pathogens ( Bozler *et al.*, 2017; Driessen *et al.*, 1989; Gillis & Hardy, 1997; Pannebakker *et al.*, 2008). The *D. phalerata* genome was first sequenced, but not assembled, in 2019 using short-read data for a study of immune gene evolution ( Hill *et al.*, 2019).

Here we present a chromosomally complete genome sequence for *Drosophila phalerata,* derived from the DNA of two male offspring from a wild female collected on a sulphur tuft fungus ( *Hypholoma fasciculare*) on the Penns in the Rocks estate, East Sussex, as part of the Darwin Tree of Life Project. This genome sequence is proving useful for resolving relationships among the Drosophilidae ( Kim *et al.*, 2023), and will further build on the value of this family as a model clade for comparative genomics and molecular evolution. This project is a collaborative effort to sequence all named eukaryotic species in the Atlantic Archipelago of Britain and Ireland.

## Genome sequence report

The genome was sequenced from one male *Drosophila phalerata* ( Figure 1) reared at the Institute of Ecology and Evolution, University of Edinburgh. A total of 111-fold coverage in Pacific Biosciences single-molecule HiFi long reads was generated. Primary assembly contigs were scaffolded with chromosome conformation Hi-C data. Manual assembly curation corrected 112 missing joins or mis-joins and removed 10 haplotypic duplications, reducing the assembly length by 1.84% and the scaffold number by 16.11%, and increasing the scaffold N50 by 7.91%.

**Figure 2.**
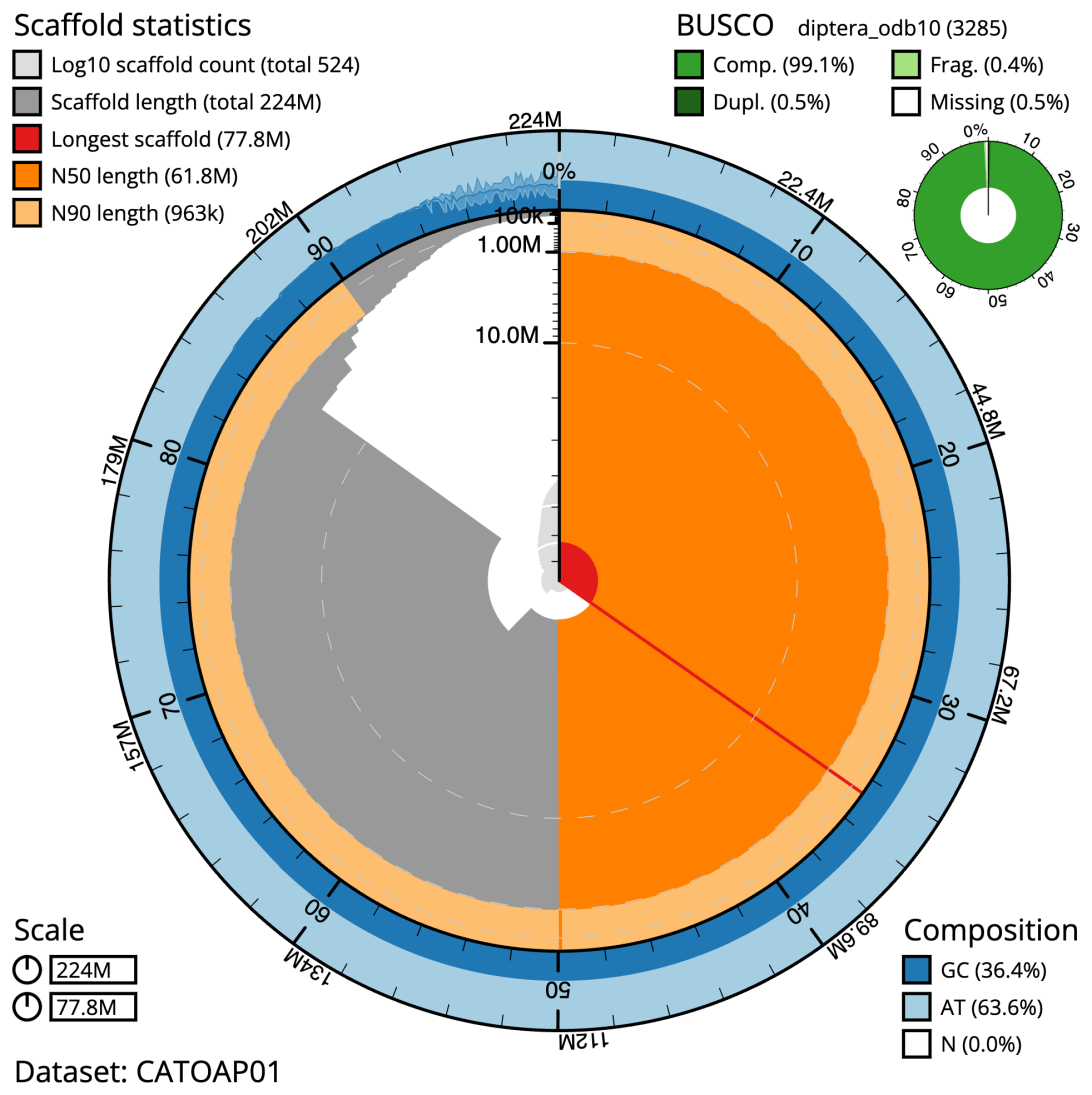
Genome assembly of *Drosophila phalerata*, idDroPhal2.2: metrics. The BlobToolKit Snailplot shows N50 metrics and BUSCO gene completeness. The main plot is divided into 1,000 size-ordered bins around the circumference with each bin representing 0.1% of the 223,932,721 bp assembly. The distribution of scaffold lengths is shown in dark grey with the plot radius scaled to the longest scaffold present in the assembly (77,813,368 bp, shown in red). Orange and pale-orange arcs show the N50 and N90 scaffold lengths (61,822,819 and 962,970 bp), respectively. The pale grey spiral shows the cumulative scaffold count on a log scale with white scale lines showing successive orders of magnitude. The blue and pale-blue area around the outside of the plot shows the distribution of GC, AT and N percentages in the same bins as the inner plot. A summary of complete, fragmented, duplicated and missing BUSCO genes in the diptera_odb10 set is shown in the top right. An interactive version of this figure is available at https://blobtoolkit.genomehubs.org/view/CATOAP01/dataset/CATOAP01/snail.

**Figure 3.**
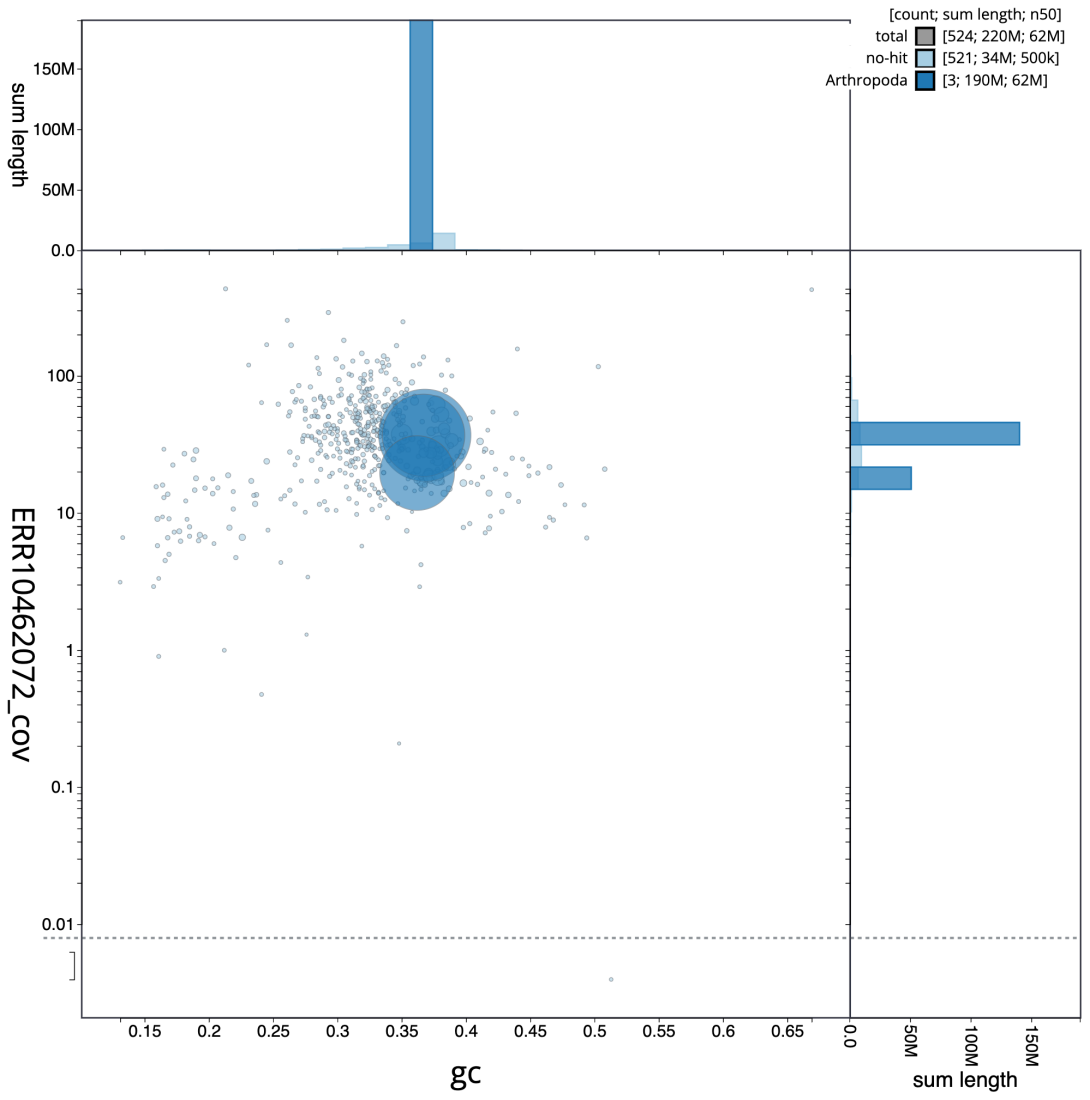
Genome assembly of *Drosophila phalerata*, idDroPhal2.2: BlobToolKit GC-coverage plot. Scaffolds are coloured by phylum. Circles are sized in proportion to scaffold length. Histograms show the distribution of scaffold length sum along each axis. An interactive version of this figure is available at https://blobtoolkit.genomehubs.org/view/CATOAP01/dataset/CATOAP01/blob.

**Figure 4.**
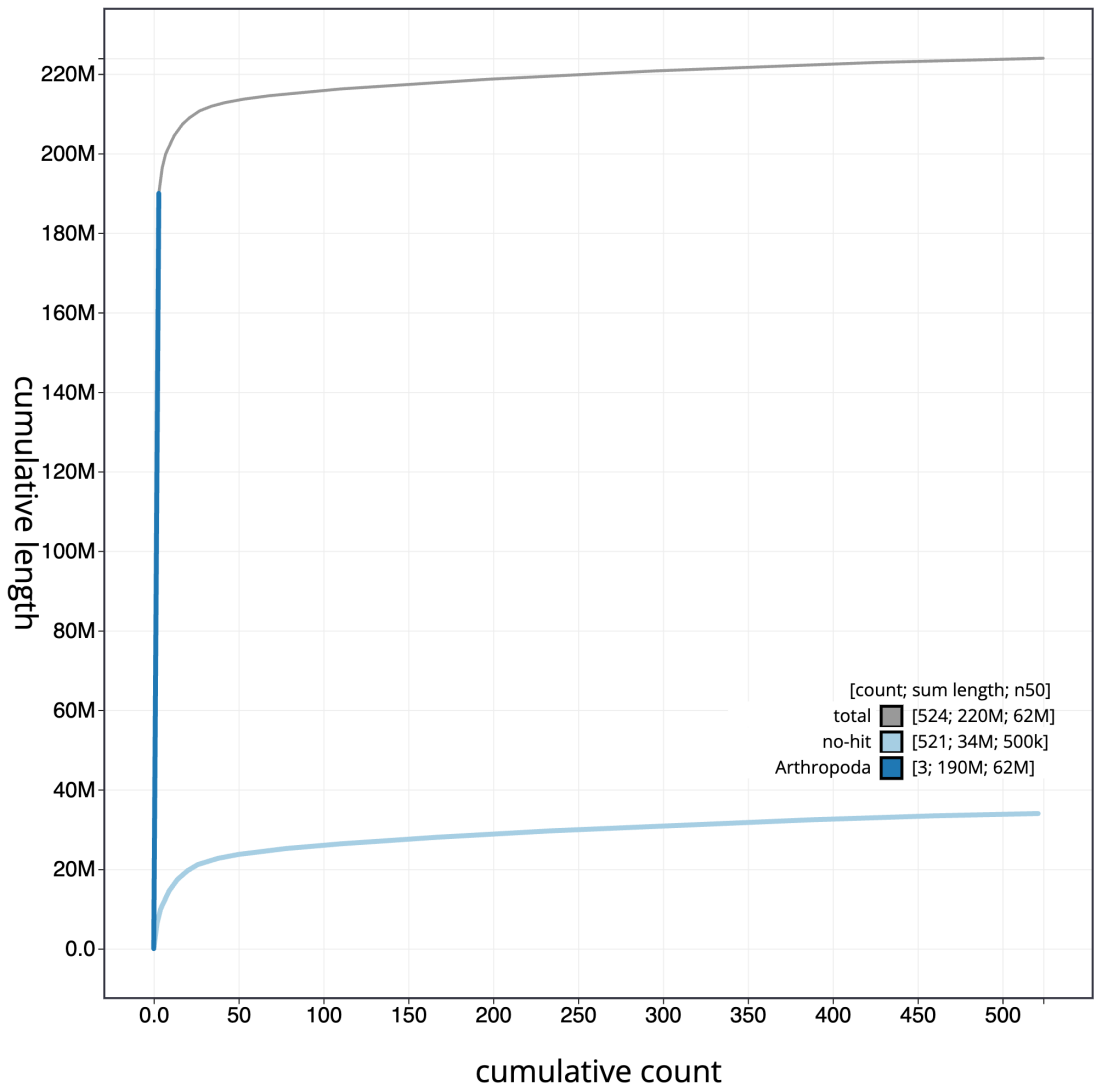
Genome assembly of *Drosophila phalerata*, idDroPhal2.2: BlobToolKit cumulative sequence plot. The grey line shows cumulative length for all scaffolds. Coloured lines show cumulative lengths of scaffolds assigned to each phylum using the buscogenes taxrule. An interactive version of this figure is available at https://blobtoolkit.genomehubs.org/view/CATOAP01/dataset/CATOAP01/cumulative.

**Figure 5.**
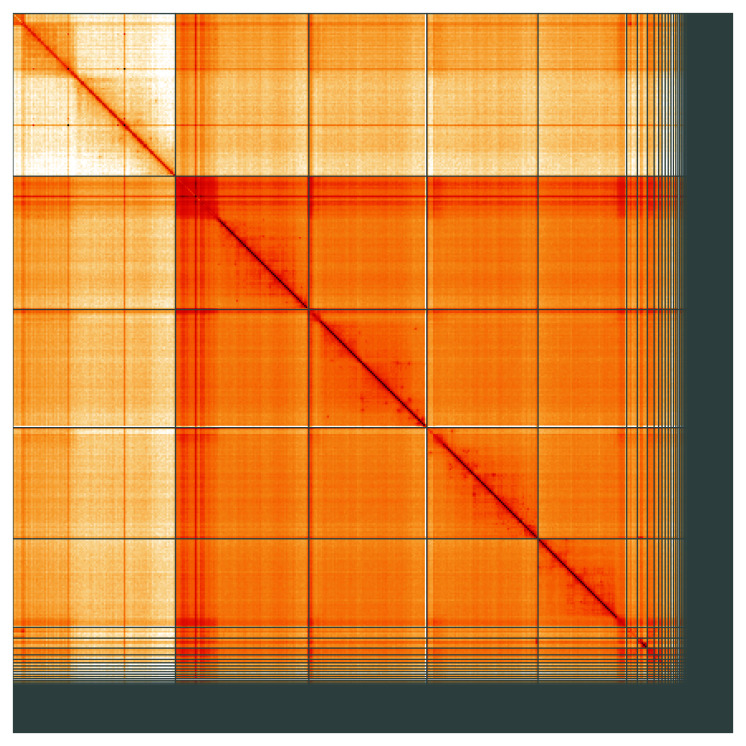
Genome assembly of *Drosophila phalerata*, idDroPhal2.2: Hi-C contact map of the idDroPhal2.2 assembly, visualised using HiGlass. Chromosomes are shown in order of size from left to right and top to bottom. An interactive version of this figure may be viewed at https://genome-note-higlass.tol.sanger.ac.uk/l/?d=I0rbqGqnTLC2Q5kb_VqYOg.

The final assembly has a total length of 223.9 Mb in 525 sequence scaffolds with a scaffold N50 of 36.5 Mb ( Table 1). The snailplot in Figure 2 provides a summary of the assembly statistics, while the distribution of assembly scaffolds on GC proportion and coverage is shown in Figure 3. The cumulative assembly plot in Figure 4 shows curves for subsets of scaffolds assigned to different phyla. Most (96.01%) of the assembly sequence was assigned to 7 chromosomal-level scaffolds, representing 5 autosomes and the X and Y sex chromosomes. Chromosome-scale scaffolds confirmed by the Hi-C data are named in order of size ( Figure 5; Table 2). The X chromosome was assigned by half coverage and synteny to *Drosophila innubila* (GCA_004354385.2) ( Hill *et al.*, 2019). Chromosome Y was identified by half-coverage. While not fully phased, the assembly deposited is of one haplotype. Contigs corresponding to the second haplotype have also been deposited. The mitochondrial genome was also assembled and can be found as a contig within the multifasta file of the genome submission.

**Table 1.** Genome data for *Drosophila phalerata*, idDroPhal2.2.

Project accession data		
Assembly identifier	idDroPhal2.2	
Species	*Drosophila phalerata*	
Specimen	idDroPhal2	
NCBI taxonomy ID	7283	
BioProject	PRJEB57266	
BioSample ID	SAMEA12110457	
Isolate information	idDroPhal2, male: whole organism (DNA sequencing) idDroPhal1, male: whole organism (Hi-C sequencing)	
Assembly metrics *		*Benchmark*
Consensus quality (QV)	65.1	*≥ 50*
*k*-mer completeness	100.0%	*≥ 95%*
BUSCO **	C:99.1%[S:98.6%,D:0.5%],F:0.4%, M:0.5%,n:3,285	*C ≥ 95%*
Percentage of assembly mapped to chromosomes	96.01%	*≥ 95%*
Sex chromosomes	XY	*localised homologous pairs*
Organelles	Mitochondrial genome: 16.14 kb	*complete single alleles*
Raw data accessions		
PacificBiosciences SEQUEL II	ERR10462072	
Hi-C Illumina	ERR10466806	
Genome assembly		
Assembly accession	GCA_951394115.2	
*Accession of alternate haplotype*	GCA_951394015.2	
Span (Mb)	223.9	
Number of contigs	894	
Contig N50 length (Mb)	0.9	
Number of scaffolds	525	
Scaffold N50 length (Mb)	36.5	
Longest scaffold (Mb)	52.6	
Genome annotation		
Number of protein-coding genes	18,973	
Number of gene transcripts	19,808	

* Assembly metric benchmarks are adapted from column VGP-2020 of “Table 1: Proposed standards and metrics for defining genome assembly quality” from ( Rhie *et al.*, 2021). ** BUSCO scores based on the diptera_odb10 BUSCO set using version 5.3.2. C = complete [S = single copy, D = duplicated], F = fragmented, M = missing, n = number of orthologues in comparison. A full set of BUSCO scores is available at https://blobtoolkit.genomehubs.org/view/CATOAP01/dataset/CATOAP01/busco.

**Table 2.** Chromosomal pseudomolecules in the genome assembly of *Drosophila phalerata*, idDroPhal2.

INSDC accession	Chromosome	Length (Mb)	GC%
OX596004.2	1	41.28	36.5
OX596005.2	2	36.53	37.0
OX596008.2	3	34.41	36.5
OY729850.1	4	27.41	37.0
OY729851.1	5	3.09	35.0
OX596006.1	X	50.32	36.0
OX596007.1	Y	3.33	38.0
OX596009.1	MT	0.02	22.0

The estimated Quality Value (QV) of the final assembly is 65.1 with *k*-mer completeness of 100.0%, and the assembly has a BUSCO v5.3.2 completeness of 99.1% (single = 98.6%, duplicated = 0.5%), using the diptera_odb10 reference set ( *n* = 3,285).

Metadata for specimens, barcode results, spectra estimates, sequencing runs, contaminants and pre-curation assembly statistics are given at https://links.tol.sanger.ac.uk/species/7283.

## Genome annotation report

The *Drosophila phalerata* genome assembly (GCA_951394115.1) was annotated using the Ensembl rapid annotation pipeline ( Table 1; https://rapid.ensembl.org/Drosophila_phalerata_GCA_951394115.1/Info/Index). The resulting annotation includes 19,808 transcribed mRNAs from 18,973 protein-coding genes.

## Methods

### Sample acquisition and nucleic acid extraction

The *Drosophila phalerata* specimens were first-generation male progeny from a wild-collected female. The sequenced flies were reared on laboratory *Drosophila* medium supplemented with ~2cm ^3^ of commercial mushroom ( *Agaricus bisporus*) to encourage egg laying. The mother was collected from sulphur tuft fungus ( *Hypholoma fasciculare*) in Penns in the Rocks Estate, East Sussex, England (latitude 51.10, longitude 0.17) on 2021-09-05. The flies were collected and identified by Darren Obbard (University of Edinburgh), and species identification was confirmed by examination of the progeny. Flies were reared at the University of Edinburgh, Scotland, and were harvested on 2021-10-02. Each living anaesthetised fly was placed directly into the collection tube and frozen from live at –80°C. The sample with specimen ID SAN00001906 (ToLID idDroPhal2) was used for DNA sequencing and the sample with specimen ID SAN00001905 (ToLID idDroPhal) was used for Hi-C scaffolding.

The workflow for high molecular weight (HMW) DNA extraction at the Wellcome Sanger Institute (WSI includes a sequence of core procedures: sample preparation; sample homogenisation, DNA extraction, fragmentation, and clean-up. In sample preparation, the idDroPhal2 sample was weighed and dissected on dry ice ( Jay *et al.*, 2023). The whole organism was homogenised using a PowerMasher II tissue disruptor ( Denton *et al.*, 2023a). HMW DNA was extracted using the Manual MagAttract v1 protocol ( Strickland *et al.*, 2023b). The DNA was then sheared into an average fragment size of 12–20 kb in a Megaruptor 3 system with speed setting 30 ( Todorovic *et al.*, 2023). Sheared DNA was purified by solid-phase reversible immobilisation ( Strickland *et al.*, 2023a): in brief, the method employs a 1.8X ratio of AMPure PB beads to sample to eliminate shorter fragments and concentrate the DNA. The concentration of the sheared and purified DNA was assessed using a Nanodrop spectrophotometer and Qubit Fluorometer and Qubit dsDNA High Sensitivity Assay kit. Fragment size distribution was evaluated by running the sample on the FemtoPulse system.

Protocols developed by the WSI Tree of Life core laboratory are available on protocols.io ( Denton *et al.*, 2023b).

### Sequencing

Pacific Biosciences HiFi circular consensus DNA sequencing libraries were constructed according to the manufacturers’ instructions. DNA sequencing was performed by the Scientific Operations core at the WSI on a Pacific Biosciences SEQUEL II instrument. Hi-C data were also generated from the whole organism tissue of idDroPhal1 using the Arima2 kit and sequenced on the Illumina NovaSeq 6000 instrument.

### Genome assembly, curation and evaluation

Assembly was carried out with HiCanu ( Nurk *et al.*, 2020) and haplotypic duplication was identified and removed with purge_dups ( Guan *et al.*, 2020). The assembly was then scaffolded with Hi-C data ( Rao *et al.*, 2014) using YaHS ( Zhou *et al.*, 2023). The assembly was checked for contamination and corrected as described previously ( Howe *et al.*, 2021). Manual curation was performed using HiGlass ( Kerpedjiev *et al.*, 2018) and Pretext ( Harry, 2022). The mitochondrial genome was assembled using MitoHiFi ( Uliano-Silva *et al.*, 2023), which runs MitoFinder ( Allio *et al.*, 2020) or MITOS ( Bernt *et al.*, 2013) and uses these annotations to select the final mitochondrial contig and to ensure the general quality of the sequence.

A Hi-C map for the final assembly was produced using bwa-mem2 ( Vasimuddin *et al.*, 2019) in the Cooler file format ( Abdennur & Mirny, 2020). To assess the assembly metrics, the *k*-mer completeness and QV consensus quality values were calculated in Merqury ( Rhie *et al.*, 2020). This work was done using Nextflow ( Di Tommaso *et al.*, 2017) DSL2 pipelines “sanger-tol/readmapping” ( Surana *et al.*, 2023a) and “sanger-tol/genomenote” ( Surana *et al.*, 2023b). The genome was analysed within the BlobToolKit environment ( Challis *et al.*, 2020) and BUSCO scores ( Manni *et al.*, 2021; Simão *et al.*, 2015) were calculated.

Table 3 contains a list of relevant software tool versions and sources.

**Table 3.** Software tools: versions and sources.

Software tool	Version	Source
BlobToolKit	4.1.7	https://github.com/blobtoolkit/blobtoolkit
BUSCO	5.3.2	https://gitlab.com/ezlab/busco
HiCanu	2.2	https://github.com/marbl/canu
HiGlass	1.11.6	https://github.com/higlass/higlass
Merqury	MerquryFK	https://github.com/thegenemyers/MERQURY.FK
MitoHiFi	2	https://github.com/marcelauliano/MitoHiFi
PretextView	0.2	https://github.com/wtsi-hpag/PretextView
purge_dups	1.2.3	https://github.com/dfguan/purge_dups
sanger-tol/genomenote	v1.0	https://github.com/sanger-tol/genomenote
sanger-tol/readmapping	1.1.0	https://github.com/sanger-tol/readmapping/tree/1.1.0
YaHS	1.2a.2	https://github.com/c-zhou/yahs

### Genome annotation

The BRAKER2 pipeline ( Brůna *et al.*, 2021) was used in the default protein mode to generate annotation for the *Drosophila phalerata* assembly (GCA_951394115.1) in Ensembl Rapid Release.

### Wellcome Sanger Institute – Legal and Governance

The materials that have contributed to this genome note have been supplied by a Darwin Tree of Life Partner. The submission of materials by a Darwin Tree of Life Partner is subject to the **‘Darwin Tree of Life Project Sampling Code of Practice’**, which can be found in full on the Darwin Tree of Life website here. By agreeing with and signing up to the Sampling Code of Practice, the Darwin Tree of Life Partner agrees they will meet the legal and ethical requirements and standards set out within this document in respect of all samples acquired for, and supplied to, the Darwin Tree of Life Project. 

Further, the Wellcome Sanger Institute employs a process whereby due diligence is carried out proportionate to the nature of the materials themselves, and the circumstances under which they have been/are to be collected and provided for use. The purpose of this is to address and mitigate any potential legal and/or ethical implications of receipt and use of the materials as part of the research project, and to ensure that in doing so we align with best practice wherever possible. The overarching areas of consideration are:

• Ethical review of provenance and sourcing of the material

• Legality of collection, transfer and use (national and international) 

Each transfer of samples is further undertaken according to a Research Collaboration Agreement or Material Transfer Agreement entered into by the Darwin Tree of Life Partner, Genome Research Limited (operating as the Wellcome Sanger Institute), and in some circumstances other Darwin Tree of Life collaborators.

## Data Availability

European Nucleotide Archive: *Drosophila phalerata*. Accession number PRJEB57266; https://identifiers.org/ena.embl/PRJEB57266 ( Wellcome Sanger Institute, 2023). The genome sequence is released openly for reuse. The *Drosophila phalerata* genome sequencing initiative is part of the Darwin Tree of Life (DToL) project. All raw sequence data and the assembly have been deposited in INSDC databases. Raw data and assembly accession identifiers are reported in Table 1.

## Author information

Members of the Darwin Tree of Life Barcoding collective are listed here: https://doi.org/10.5281/zenodo.4893703.

Members of the Wellcome Sanger Institute Tree of Life Management, Samples and Laboratory team are listed here: https://doi.org/10.5281/zenodo.10066175.

Members of Wellcome Sanger Institute Scientific Operations: Sequencing Operations are listed here: https://doi.org/10.5281/zenodo.10043364.

Members of the Wellcome Sanger Institute Tree of Life Core Informatics team are listed here: https://doi.org/10.5281/zenodo.10066637.

Members of the Tree of Life Core Informatics collective are listed here: https://doi.org/10.5281/zenodo.5013541.

Members of the Darwin Tree of Life Consortium are listed here: https://doi.org/10.5281/zenodo.4783558.
