# Supplementary material for: The genome sequence of the drosophilid fruit fly, Drosophila phalerata (Meigen, 1830)
Source: Wellcome Open Res. 2024 Feb 19;9:63. [Version 1] doi: 10.12688/wellcomeopenres.20634.1 (PMC11126896; doi:10.12688/wellcomeopenres.20634.1)
Supplement: Peer review history

# Reviewer report: Reviewer response for version 1

**DOI:** 10.21956/wellcomeopenres.22837.r83122

**Published:** 2024-05-30

**Authors:** Jerome H.L Hui

**Affiliations:** 1 The Chinese University of Hong Kong, Hong Kong, Hong Kong

Obbard and colleagues report the genome sequence of drosophild *Drosophila phalerata *(Meigen, 1830). There are records showing that this species can be found in mainland UK and Northern Ireland. The high-quality genome resource presented in this study will be very useful to understand the evolution of dipterans, the cross-kingdom interactions with fungi, the ecology and population structure of drosophilids, as well as their response to climate change. Moreover, as fruit fly *Drosophila melanogaster *is the best studied insect laboratory model in the world, the genome resource presented here for another species in the genus *Drosophila *facilitates the possibility of introducing or developing new laboratory model.

 This genome resource is excellent from the summary statistics, with high BUSCO numbers and sequence continuity, and majority of sequences contained on the 5 pseudochromosomes (plus two sex chromosomes and mitochondrion). All in all, this is a valuable contribution by the Darwin Tree of Life.

Are sufficient details of methods and materials provided to allow replication by others?

Yes

Is the rationale for creating the dataset(s) clearly described?

Yes

Are the datasets clearly presented in a useable and accessible format?

Yes

Are the protocols appropriate and is the work technically sound?

Yes

Reviewer Expertise:

Genomics, evolution, invertebrates

I confirm that I have read this submission and believe that I have an appropriate level of expertise to confirm that it is of an acceptable scientific standard.

# Reviewer report: Reviewer response for version 1

**DOI:** 10.21956/wellcomeopenres.22837.r83128

**Published:** 2024-05-24

**Authors:** Edson Ishengoma

**Affiliations:** 1 University of Dar es Salaam, Dar es Salaam, Dar es Salaam, Tanzania

This manuscript presents the assembled genome of *Drosophila phalerata,* a fruit-fly species that feeds on mushrooms. As usual, Drosophilid species have four pairs of chromosomes including the sex chromosomes, X and Y in males. The 7 chromosomal psuedomolecules including the X and Y pair that have been scaffolded during this study reflect the typical karyotype of Drosophilids. ~19K proteins that are extracted from this genome will obviously advance the ecology, evolution and functional diversity of Drosophila species.

Are sufficient details of methods and materials provided to allow replication by others?

Yes

Is the rationale for creating the dataset(s) clearly described?

Yes

Are the datasets clearly presented in a useable and accessible format?

Yes

Are the protocols appropriate and is the work technically sound?

Yes

Reviewer Expertise:

Genomics, Bioinformatics, Evolution

I confirm that I have read this submission and believe that I have an appropriate level of expertise to confirm that it is of an acceptable scientific standard.

# Reviewer report: Reviewer response for version 1

**DOI:** 10.21956/wellcomeopenres.22837.r83125

**Published:** 2024-05-17

**Authors:** Ravikumar Dodiya

**Affiliations:** 1 Sardarkrushinagar Dantiwada Agricultural University, Sardarkrushinagar, Dantiwada, India

The genomic sequencing and annotation of *Drosophila phalerata*, a fungus-breeding fruit fly species, have been meticulously detailed in the provided report. Leveraging advanced sequencing technologies, the genome was assembled to a high standard, encompassing 223.9 Mb across 525 sequence scaffolds. Notably, chromosome-level scaffolds were identified, with a significant portion of the assembly assigned to seven chromosomal-level structures, including autosomes and sex chromosomes. Annotation efforts revealed a rich genetic landscape, comprising 18,973 protein-coding genes and 19,808 transcribed mRNAs. The process involved meticulous sample acquisition, laboratory rearing, and DNA extraction, followed by thorough sequencing protocols. To ensure accuracy and completeness, the assembly underwent extensive curation and evaluation, employing bioinformatic tools and high-quality standards. Overall, this comprehensive genomic analysis significantly enhances our understanding of *Drosophila phalerata*, shedding light on its evolutionary context within the Drosophilidae family and providing a valuable resource for further research in molecular evolution and comparative genomics.

Are sufficient details of methods and materials provided to allow replication by others?

Yes

Is the rationale for creating the dataset(s) clearly described?

Yes

Are the datasets clearly presented in a useable and accessible format?

Yes

Are the protocols appropriate and is the work technically sound?

Yes

Reviewer Expertise:

Biological control, IPM

I confirm that I have read this submission and believe that I have an appropriate level of expertise to confirm that it is of an acceptable scientific standard.

# Reviewer report: Reviewer response for version 1

**DOI:** 10.21956/wellcomeopenres.22837.r80917

**Published:** 2024-05-17

**Authors:** Manee M Manee

**Affiliations:** 1 King Abdulaziz City for Science and Technology, Riyadh, Saudi Arabia

Obbard et al. generated a complete genome assembly of the drosophilid fruit fly, *Drosophila phalerata*, derived from a male specimen. The assembly is 223.9 Mb in size, with most of the genome organized into seven chromosomal pseudomolecules, including the sex chromosomes X and Y, and a fully assembled mitochondrial genome. The annotation process identified 18,973 protein-coding genes. This research enhances our understanding of the genetic structure and evolutionary biology of *Drosophila phalerata* and provides a valuable resource for further comparative genomic studies within the Drosophilidae family. Here are minor suggestions:

 1- I would ask the authors to clarify why the accession number in the text is version 1 (GCA_951394115.1) and in the table version 2 (GCA_951394115.2). It seems they updated to version 2, and they should specify which version they are reporting. Please check the parameters in Table 1 against the numbers reported in https://www.ncbi.nlm.nih.gov/datasets/genome/GCA_951394115.2/

 2- I would suggest including in the background, or any appropriate section of the manuscript, why the study of Drosophila phalerata's genome is important. For instance, mention any specific applications of this research in evolutionary biology or other fields.

Are sufficient details of methods and materials provided to allow replication by others?

Yes

Is the rationale for creating the dataset(s) clearly described?

Yes

Are the datasets clearly presented in a useable and accessible format?

Yes

Are the protocols appropriate and is the work technically sound?

Yes

Reviewer Expertise:

bioinformatics, genomics, and evolutionary biology

I confirm that I have read this submission and believe that I have an appropriate level of expertise to confirm that it is of an acceptable scientific standard, however I have significant reservations, as outlined above.
